# Elevated serum immunoglobulin level predicts high risk of 1-year recurrence in patients with Takayasu arteritis

**DOI:** 10.1186/s13075-023-03016-8

**Published:** 2023-03-07

**Authors:** Yanqiu Guo, Juan Du, Taotao Li, Na Gao, Shiyu Yang, Yaxin Zhang, Lili Pan

**Affiliations:** Department of Rheumatology and Immunology, Beijing Anzhen Hospital, Capital Medical University, No.2 Anzhen Road, Chaoyang District, Beijing, 100029 China

**Keywords:** Takayasu arteritis, Immunoglobulin, Disease activity, Recurrence

## Abstract

**Background:**

The mechanism of humoral immunity to Takayasu arteritis (TAK) is not clear. In our study, we aimed to investigate the correlation between immunoglobulins and disease activity and the relationship between immunoglobulins and the prognosis of TAK patients.

**Methods:**

One hundred ninety TAK patients divided into two groups according to whether they had elevated immunoglobulins or not. We compared the demographic data and the clinical data between the two groups. Pearson correlation was used to analyze the relationship between immunoglobulin and disease activity, as well as the relationship between their changes. Immunohistochemical staining was used to compare the expression of humoral immune cells in TAK and atherosclerotic patients. One hundred twenty TAK patients who achieved remission within 3 months after discharge were followed up for 1 year. Logistic regression was used to explore the relationship between elevated immunoglobulins and recurrence.

**Results:**

Disease activity and inflammatory factors were significantly higher in the group with elevated immunoglobulins than in the normal group [NIH (3.0 vs. 2.0, *P* = 0.001), ITAS-A (9.0 vs. 7.0, *P* = 0.006)]. Compared with atherosclerotic patients, CD138 + plasma cells were significantly increased in the aortic wall of patients with TAK (*P* = 0.021). Changes in IgG correlated well with CRP and ESR [CRP (*r* = 0.40, *P* = 0.027), ESR (*r* = 0.64, *P* < 0.001)]. For patients with TAK in remission, elevated immunoglobulins was associated with 1-year recurrence [OR95%, CI: 2.37 (1.03, 5.47), *P* = 0.042].

**Conclusions:**

Immunoglobulins is of clinical value in evaluating disease activity in TAK patients. Moreover, the dynamic changes of IgG were correlated with the changes in inflammatory indicators in TAK patients.

## Introduction

Takayasu arteritis (TAK) is a chronic, nonspecific, granulomatous vasculitis of unknown etiology that primarily involves the aorta and its primary branches [1]. Extensive inflammatory cell infiltration leads to damage to the vessel wall, resulting in stenosis, occlusion, or dilatation of the artery and aneurysm [2]. The etiology of TAK is still unclear. In the past decades, a large amount of evidence has confirmed that cell-mediated autoimmunity is closely related to the pathogenesis of TAK. A variety of cytokines, including interferon-γ (IFN-γ), tumor necrosis factor-α (TNF-α), interleukin-2 (IL-2), interleukin-6 (IL-6), interleukin-12 (IL-12), interleukin-8 (IL-8), interleukin-17A (IL-17A), and interleukin-18 (IL-18), were higher in TAK patients than those in healthy controls and were related to disease activity [3–7]. However, the involvement of humoral immunity in the pathogenesis of TAK is still controversial [8, 9].

It was demonstrated that in patients with active TAK, there was B cell infiltration in the outer membrane of the artery, an increase in the number of B cells in the peripheral blood, and an increase in the level of B cell activating factor. And it has been shown by studies that rituximab is effective in the treatment of refractory aortitis [10–12]. In recent years, it has been found that purified IgG from TAK patients can specifically activate the mTOR pathway in endothelial cells and participate in the pathogenesis of TAK by causing vascular remodeling [13]. These all suggest that B cells also play an important role in the pathogenesis of Takayasu arteritis.

To provide more clinical evidence for the association between humoral immunity and TAK, this study reviewed and summarized the clinical and serological characteristics of TAK patients with elevated immunoglobulins, analyzed the correlation between immunoglobulin and disease activity, and evaluated the relationship between immunoglobulin and the prognosis of TAK patients.

## Methods

### Study population and grouping

This study retrospectively enrolled 190 TAK patients admitted to the Department of Rheumatology and Immunology, Beijing Anzhen Hospital, from October 2014 to June 2021. All patients were diagnosed with TAK according to the criteria for the classification of TAK developed by the American College of Rheumatology in 1990 [14]. Patients with other autoimmune diseases, liver and kidney dysfunction, cancer, or infections were excluded from the study.

Disease activity was assessed using a modified version of Kerr’s criteria [NIH (National Institutes of Health) score]; ITAS-A (Indian Takayasu’s Arteritis Activity Score with acute-phase reactants), and ITAS2010 (Indian Takayasu’s Arteritis Activity Score) [15, 16]. One item of IgG, immunoglobulin (IgA), or immunoglobulin (IgM) is higher than the normal range [IgA (g/L): 1.0–4.2, IgG (g/L): 8.4–17.4, IgM (g/L): 0.3–2.2] which was defined as the elevated immunoglobulin group. Immunoglobulins were detected by an automatic analyzer (Hitachi 7600–120, Tokyo, Japan).

This retrospective study was conducted following the ethical principles of the Declaration of Helsinki and approved by the Ethics Committee of Beijing Anzhen Hospital, Capital Medical University (number:2022244X).

### Collection of clinical data

Data regarding age, sex, body mass index (BMI), disease duration, and clinical manifestation were recorded at baseline. Lesions were classified according to the angiographic classification of the 1994 International TAK Conference in Tokyo. Laboratory data were obtained from laboratory examination reports of the participating hospital. Numano classification of 1996 was adopted for image classification, divided into six types. According to the Numano standard, the imaging types of the patients were divided into six types (I, IIa, IIb, III, IV, and V) [17].

### Method of pathology

We collected surgically resected vessel specimens from four TAK and three atherosclerotic patients from the Beijing Anzhen Hospital from 2018 to 2020.

We processed the samples using immunohistochemical staining. Aortic specimens were fixed in 4% neutral formalin for 24 h, embedded in paraffin, and sectioned (4 μm). Then, they were stained with primary antibody for CD138 (Item No.ab46506, Abcam, Cambridge, CB2 0AX, UK) overnight at 4 °C and secondary antibody for 0.5–1 h at room temperature and detected with 3,3′-diaminobenzidine. Antibodies were diluted with triple-buffered saline (TBS) solution containing 1% bovine serum albumin (BSA).

A Nikon microscope eclipse 90i (Nikon, Tokyo, Japan) was used for image capture and analysis by the NIS-Elements BR 3.1 software (Nikon). Positive areas for immunohistochemical staining were measured in 8–10 areas of each section, and the mean value was expressed as a percentage of the total area.

### Follow-up and outcome

Disease activity was assessed at 1, 3, and 6 months after discharge and every 6 months after that, and 130 of the 190 patients completed our follow-up. Of the 130 patients, 120 achieved remission within 3 months after discharge.

Remission was defined as the following: no clinical signs and symptoms of active TAK, normal ESR and CRP levels, and no evidence of progressive stenosis or dilatation in the involved vessels [18].

The outcome of this study was disease 1-year recurrence. Recurrence was defined as having any of the following conditions: (a) clinical features of ischemia (such as stroke and limb claudication) and (b) evidence of active aortic inflammation leading to progression of vascular involvement [18].

### Statistical analysis

Normally distributed continuous variables were expressed as mean ± standard deviation (SD), and comparison between groups was analyzed by ANOVA. Skewed data were expressed as the median and interquartile range (IQR) and compared with the Kruskal–Wallis test. Categorical variables were expressed as numbers (percentage), and the chi-square test was used to compare between groups.

Correlation analysis (Pearson correlation analysis) was used to determine the relationship between increased levels of immunoglobulin and disease activity (NIH, ITAS-A, ITAS2010) and the relationship between changes in immunoglobulin and changes in disease activity and inflammatory markers. The results are expressed as correlation coefficients (*r*) and *P* value (*P*).

The comparison of positive areas for immunohistochemical staining in the TAK group and healthy controls was performed using the *t*-test.

In this study, logistic regression was used to evaluate the association between the elevated immunoglobulin and 1-year recurrence. Sex, age, duration of TAK, and BMI were adjusted as covariates.

All tests were bilateral, and *P* < 0.05 was considered statistically significant. All data analyses were performed by the R software (R-project ®; R Foundation for Statistical Computing, Vienna, Austria, ver. 4.2.1).

## Results

### Comparison of baseline characteristics between the elevated immunoglobulin group and the normal group in all 190 TAK patients

Among the 190 TAK patients, 43 cases (22.63%) had elevated immunoglobulin. There was no difference in demographic indexes such as sex, age, duration of disease, and BMI between the two groups. Patients with normal immunoglobulin had a bigger proportion of claudication (21.8% vs. 7.0%, *P* = 0.028), but there was no significant difference in the remaining symptoms. Patients with elevated immunoglobulin had a higher level of ESR (34.0 vs. 14.0 mm/h, *P* < 0.001), CRP (12.5 vs. 2.4 mg/L, *P* < 0.001), and C3 (1.3 ± 0.2 vs. 1.1 ± 0.3 g/L, *P* = 0.004) than patients with normal immunoglobulin.However, the two groups did not differ in laboratory tests such as WBC, C4, and B lymphocytes (Table 1).

**Table 1:** Baseline characteristics of participants with all 190 TAK patients

	**Normal immunoglobulin** **(*N* = 147)**	**Elevated immunoglobulin** **(*N* = 43)**	***P*-value**
**Demographic data**			
Age, years	38.6 ± 13.0	37.3 ± 10.6	0.549
Female, *n* (%)	131 (89.1%)	40 (93.0%)	0.452
BMI, kg/m^2^	22.4 ± 3.1	22.0 ± 5.1	0.526
Duration of TAK, months	60.0 (12.0–156.0)	60.0 (12.0–120.0)	0.290
**Clinical symptoms**			
Fever, *n* (%)	20 (13.6%)	5 (11.6%)	0.736
Fatigue, *n* (%)	63 (42.9%)	20 (46.5%)	0.671
Claudication, *n* (%)	32 (21.8%)	3 (7.0%)	0.028
Blood pressure asymmetry in both upper limbs, *n* (%)	56 (38.1%)	16 (37.2%)	0.916
Neck pain, *n* (%)	21 (14.3%)	3 (7.0%)	0.204
Chest distress, *n* (%)	39 (26.5%)	13 (30.2%)	0.632
Chest pain, *n* (%)	31 (21.1%)	9 (20.9%)	0.982
Dizzy, *n* (%)	73 (49.7%)	17 (39.5%)	0.242
Headache, *n* (%)	32 (21.8%)	7 (16.3%)	0.433
**Laboratory tests**			
WBC, × 10^9^/L	7.4 ± 3.0	7.7 ± 2.3	0.544
ESR, mm/h	14.0 (7.0–24.0)	34.0 (18.0–67.0)	< 0.001
CRP, mg/L	2.4 (0.6–8.6)	12.5 (2.2–28.4)	< 0.001
C3, g/L	1.1 ± 0.3	1.3 ± 0.2	0.004
C4, g/L	0.2 (0.2–0.3)	0.2 (0.2–0.3)	0.408
B lymphocyte	212.0 (151.0–357.0)	259.0 (182.0–319.0)	0.332
B lymphocyte, %	13.4 ± 6.2	13.8 ± 5.2	0.739
Creatinine, µmol/L	54.0 (47.0–64.5)	56.2 (48.3–64.8)	0.432
Aspartate aminotransferase, U/L	17.0 (13.5–21.0)	16.0 (12.2–19.7)	0.103
**Imaging type**			
I, *n* (%)	18, 12.24%	11, 25.59%	0.032
IIa, *n* (%)	9, 6.12%	1, 2.33%	0.553
IIb, *n* (%)	18, 12.24%	8, 18.60%	0.286
III, *n* (%)	7, 4.76%	2, 4.65%	1.000
IV, *n* (%)	5, 3.40%	1, 2.33%	1.000
V, *n* (%)	90, 61.22%	20, 46.51%	0.086
**Disease activity**			
NIH score	2.0 (2.0–3.0)	3.0 (2.0–3.0)	0.001
ITAS-A score	7.0 (3.0–10.0)	9.0 (6.0–12.0)	0.006
ITAS2010 score	6.0 (3.0–9.0)	7.0 (3.5–9.0)	0.267

*Abbreviations*: *BMI* body mass index, *TAK* Takayasu’s arteritis, *WBC* white blood cell, *ESR* erythrocyte sedimentation rate, *CRP* C reactive protein, *NIH score* National Institutes of Health score, *ITAS-A* Indian Takayasu’s Arteritis Activity Score with acute-phase reactants, *ITAS2010* Indian Takayasu’s Arteritis Activity Score

Among patients with elevated immunoglobulin, the proportion of Numano type I was significantly higher than the normal immunoglobulin group (25.59% vs. 12.24%, *P* = 0.032). There was no significant difference in the proportion of other types between the two groups. We also analyzed the disease activity of the two groups and found the proportion of elevated NIH (3.0 vs.2.0, *P* = 0.001) and ITAS-A (9.0 vs.7.0, *P* = 0.006) scores in the elevated immunoglobulin group was significantly higher than in the normal immunoglobulin group. However, there was no difference in the ITAS2010 score between the two groups (Table 1).

### Characteristics of the elevated immunoglobulin group and the correlation of immunoglobulin and disease activity

**Fig. 1:**
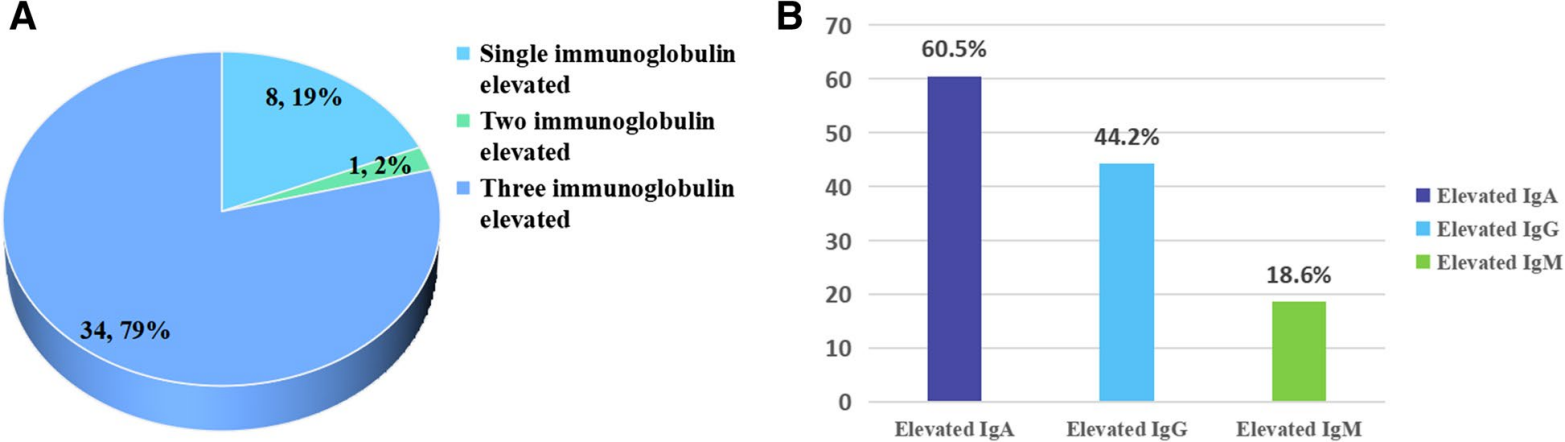
Characteristics of elevated immunoglobulin group. **A** The proportion of single, two, and three elevated immunoglobulins. **B** The proportion of three elevated IgA, IgG, and IgM

As shown in Fig. 1A, among 190 TA patients, most patients had increased single immunoglobulin (34 cases), and a few had increased two (8 cases) or three immunoglobulins (1 case). As shown in Fig. 1B, 43 patients had elevated immunoglobulin, mainly IgG (26/43), followed by IgA (19/43), and at least IgM (8/43) (Fig. 1).

**Fig. 2:**
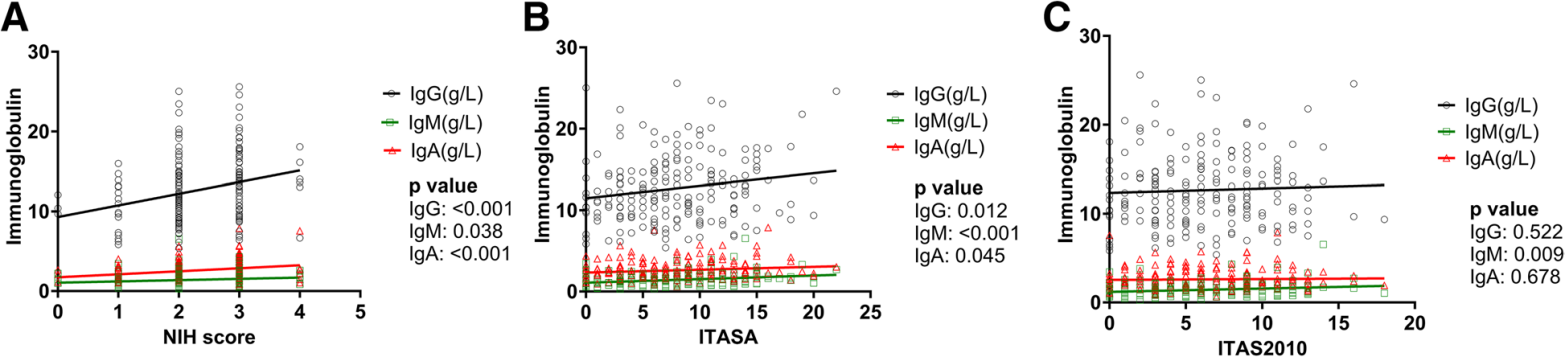
Correlation between immunoglobulin and disease activity in all 190 TAK patients. **A** Correlation between immunoglobulin and NIH in all 190 TAK patients. **B** Correlation between immunoglobulin and ITASA in all 190 TAK patients. **C** Correlation between immunoglobulin and ITAS2010 in all 190 TAK patients

We calculated the correlation between immunoglobulins and disease activity in 190 TAK patients. IgM was positively correlated with NIH (*r* = 0.15, *P* = 0.038), ITAS-A (*r* = 0.27, *P* < 0.001), and ITAS2010 (*r* = 0.19, *P* = 0.009). IgA was positively correlated with NIH (*r* = 0.24, *P* < 0.001) and ITAS-A (*r* = 0.15, *P* = 0.045) but was not correlated with ITAS2010 (*r* = 0.03, *P* = 0.678). IgG was positively correlated with NIH (*r* = 0.26, *P* < 0.001) and ITAS-A (*r* = 0.18, *P* = 0.012) but was not correlated with ITAS2010 (*r* = 0.05, *P* = 0.522) (Table 2, Fig. 2).

**Table 2:** Correlation between immunoglobulin and disease activity in all 190 TAK patients

	**NIH score** ***r*, *P* value**	**ITAS-A score** ***r*, *P* value**	**ITAS2010 score** ***r*, *P* value**
**IgM**	0.15, 0.038	0.27, < 0.001	0.19, 0.009
**IgA**	0.24, < 0.001	0.15, 0.045	0.03, 0.678
**IgG**	0.26, < 0.001	0.18, 0.012	0.05, 0.522

*Abbreviations*: *NIH score* National Institutes of Health score, *ITAS-A* Indian Takayasu’s Arteritis Activity Score with acute-phase reactants, *ITAS2010* Indian Takayasu’s Arteritis Activity Score

### The quantification of CD138 staining in TAK (*n* = 4) and control group (*n* = 3)

**Fig. 3:**
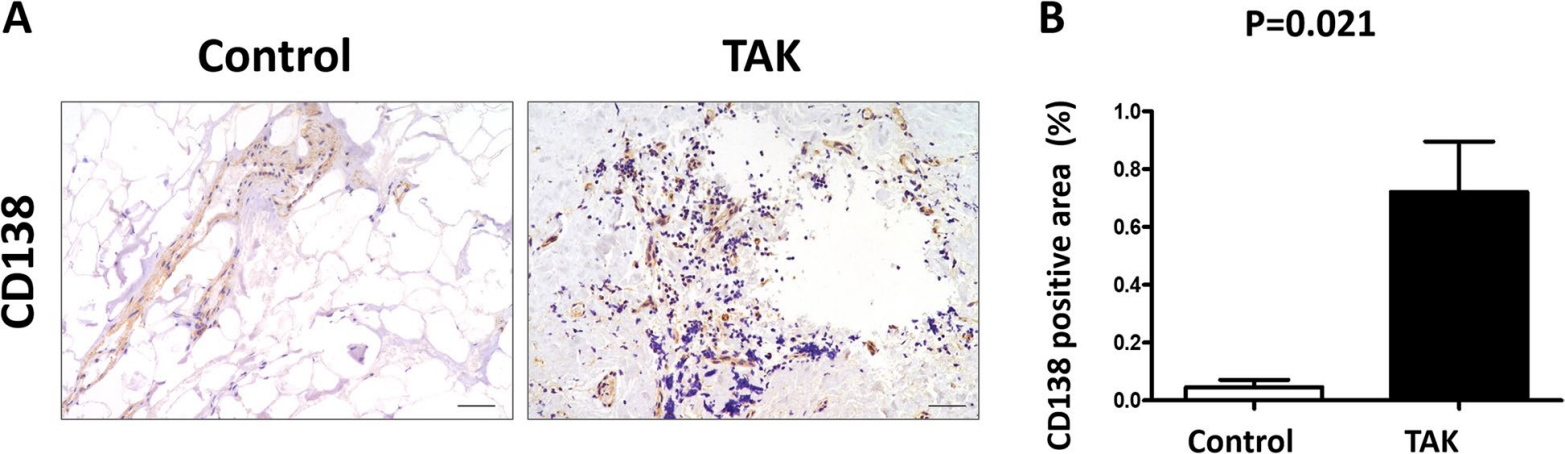
CD138 expressions in the aortic wall of TAK and control group. **A** CD138 staining in the aortic wall of TAK patients and control group (scale bars, 100 μm; *n* = 3 in control group, *n* = 4 in TAK group). **B** The quantification of CD138 staining in the two groups (*n* = 3 in control group, *n* = 4 in TAK group)

We detected the expressions of CD138 + plasma cells in the aortic wall of TAK (*n* = 4) and atherosclerosis patients (*n* = 3). Immunohistochemical staining showed the increased infiltration of CD138 + plasma cells (72.08% ± 34.97% vs 4.63% ± 4.37%; *P* = 0.021) in the aortic tissue of TAK patients, compared with the atherosclerosis patients (Fig. 3).

### Correlation between immunoglobulin change and disease activity and inflammatory indicators change in 31 elevated immunoglobulin group during the follow-up

**Fig. 4:**
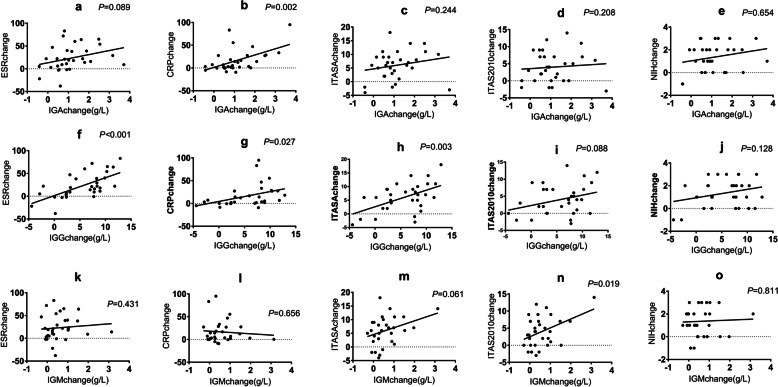
Correlation between immunoglobulin change and disease activity in 31 elevated immunoglobulin group during the follow-up. **a**–**e** Correlation between IgA change and disease activity and inflammatory indicators change in 31 elevated immunoglobulin group during the follow-up. **f**–**j** Correlation between IgG change and disease activity and inflammatory indicators change in 31 elevated immunoglobulin group during the follow-up. **k**–**o** Correlation between IgM change and disease activity and inflammatory indicators change in 31 elevated immunoglobulin group during the follow-up

In these 31 patients, we calculated the change in immunoglobulin, disease activity score, CRP, and ESR. The change in IgM was positively correlated with the change in ITAS2010 (*r* = 0.42, *P* = 0.019) and ITAS-A (*r* = 0.637, *P* = 0.008) but was not correlated with the change in NIH (*r* = 0.04, *P* = 0.811), ITAS-A (*r* = 0.34, *P* = 0.061), CRP (*r* =  − 0.08, *P* = 0.656), and ESR (*r* = 0.09, *P* = 0.431). The change in IgA was positively correlated with the change in CRP (*r* = 0.53, *P* = 0.002) but was not correlated with other indicators. The change in IgG was positively correlated with the change in ITAS-A (*r* = 0.52, *P* = 0.003), CRP (*r* = 0.40, *P* = 0.027), and ESR (*r* = 0.64, *P* < 0.001) but was not correlated with the change in NIH (*r* = 0.28, *P* = 0.128) and ITAS2010 (*r* = 0.31, *P* = 0.088) (Table 3, Fig. 4).

**Table 3:** Correlation between immunoglobulin change and disease activity and inflammatory indicators change in 31 elevated immunoglobulin group during the follow-up

	**NIH score change** ***r*, *P* value**	**ITAS-A score change** ***r*, *P* value**	**ITAS2010 score change** ***r*, *P* value**	**CRP change** ***r*, *P* value**	**ESR change** ***r*, *P* value**
**IgM change**	0.04, 0.811	0.34, 0.061	0.42, 0.019	-0.08, 0.656	0.09, 0.431
**IgA change**	0.23, 0.208	0.22, 0.244	0.08, 0.654	0.53, 0.002	0.31, 0.089
**IgG change**	0.28, 0.128	0.52, 0.003	0.31, 0.088	0.40, 0.027	0.64, < 0.001

*Abbreviations*: *CRP* C reactive protein, *NIH score* National Institutes of Health score, *ITAS-A* Indian Takayasu’s Arteritis Activity Score with acute-phase reactants, *ITAS2010* Indian Takayasu’s Arteritis Activity Score

### The relationship between elevated immunoglobulin and 1-year recurrence

One hundred twenty TAK patients who achieved remission within 3 months after discharge were followed up for 1 year. As shown in Table 4, the risk of 1-year recurrence was 2.37 times higher in the elevated immunoglobulin group than in the normal immunoglobulin group [OR 95%; CI: 2.37 (1.03, 5.47), *P* = 0.042]. Further analysis by immunoglobulin subgroup showed that the risk of 1-year recurrence was 2.97 times higher in the elevated IgG group than in the normal immunoglobulin group [OR 95%; CI: 2.97 (1.12, 7.86), *P* = 0.028], but no similar results were found in the elevated IgA and IgM groups(*P* both > 0.05) (Table 4).

**Table 4:** The relationship between elevated immunoglobulin and 1-year recurrence

	**OR (95%CI)**	***P* value**
**Normal immunoglobulin**	Reference	
**Elevated immunoglobulin**	2.37 (1.030, 5.470)	0.042
**Elevated IgM**	1.58 (0.282, 8.846)	0.603
**Elevated IgA**	2.13 (0.606, 7.504)	0.238
**Elevated IgG**	2.97 (1.121, 7.864)	0.028

Logistic regression was used and adjusted following covariates sex, age, duration of TAK, and BMI

## Discussion

This study found that increased immunoglobulin was associated with increased disease activity in TAK patients. CD138 + plasma cells were significantly increased in the aortic wall of TAK patients, and immunoglobulin had a specific clinical value in evaluating disease activity in TAK patients. This study also found that the changes in immunoglobulin were poorly correlated with the changes in disease activity, but the changes in IgG were well correlated with CRP and ESR. For patients with TAK in remission, elevated immunoglobulin was associated with 1-year recurrence.

Most previous studies have explored the correlation between T cell-related indicators and TAK [3–7], but few have explored the role of B cell-related indicators. Even the role of B cells in TAK is controversial. Our previous study found that enhanced B cells may contribute to the pathogenesis of TAK [19]. Serum IgG levels could be used as a simple and useful biomarker to assess the disease activity of TAK and to monitor the response to therapy. In terms of clinical studies, few studies have explored the relationship between B cell-related indicators and activity and prognosis of TAK. Chen Rongyi et al. [20] found that IgG and IgA elevated significantly in active TAK patients and had a predictive value for disease activity (AUC of IgG: 0.617 (0.549–0.685); AUC of IgA: 0.592 (0.524–0.661)). Bimba F Hoyer et al. [10] found the level of CD19 + /CD20 − /CD27 antibody-secreting cells, produced by B cells, in the peripheral blood of active TAK patients was higher than it of healthy populations. Moreover, Abhishek Zanwar et al. evaluated serum levels of B cell survival factors activation factor (BAFF) and a proliferation-inducing ligand (APRIL) in TAK patients. They found APRIL was higher in active TAK patients than inactive patients [11]. Consistent with previous studies, our study also found that B cell-related factors (immunoglobulins) were correlated with disease activity in TAK patients. In addition, this study adopted more indicators (NIH, ITATS, and ITAT2010) to evaluate disease activity. In previous studies, only a single evaluation indicator was used to evaluate disease activity only by NIH [10, 20] or ITATS [11]. This study found that different types of immunoglobulins correlated with disease activity assessed by NIH score and ITATS score but had a poor correlation with the ITAS2010 score.

The role of humoral immunity has also been found in recent studies in the pathophysiological process of TAK [9, 21]. Serum immunoglobulin is secreted by plasma cells activated by B cells and plays an important role in humoral immunity. As early as the 1960s, Japanese scholars detected the existence of anti-aortic antibodies in the serum of TAK patients, and the detection rate was high in active TAK patients [22]. However, the corresponding antigen of aortic antibodies could not be defined, and the repeatability was poor. Later studies gradually found that a variety of autoantibodies, such as anti-endothelial cell autoantibody (AECA), anti-cardiolipin antibody (ACL), β2 glycoprotein I antibody (anti-β2GPI), anti-annexin V antibody (AAVA), and other antibodies had a high correlation with TAK [23, 24], but all of them lacked specificity. Some scholars have also found that rituximab B cell depletion therapy is effective in treating some TAK patients and proposed the role of serum IgG in the progression of TAK disease [10, 25]. Similarly, our study also found that TAK patients with elevated immunoglobulin had higher disease activity, considering that B cell activation plays an important role in the pathogenesis of aortitis. S J Inder et al. [26] found that B cell and plasma cell infiltration were also observed in the pathological specimens of the aortic wall of TAK patients. The pathology we studied showed the same results; CD138 + plasma cells were significantly increased in the aortic wall of TAK patients. This provides more clinical basis for the promoting role of humoral immunity in TAK. Moreover, the disorder of B lymphocyte subsets was also detected in the serum of TAK patients, and the number of plasma cells was positively correlated with disease activity [10]. However, in our study, there was no difference in the number of B lymphocytes between the elevated immunoglobulin group and the normal immunoglobulin group of TAK patients. It may be because the number of B cells is the same, but the function is changed. We found that patients with elevated immunoglobulins had higher levels of C3 values and a greater proportion of type I. It has been reported in the literature that the levels of complement and immunoglobulin will increase during TAK disease activity, and C3 can effectively evaluate the disease activity of TAK [20]. This may be because most TAK patients who come to our hospital are young women who are first-time patients and have disease activity. Usually, the degree of inflammation is high, but the inflammatory infiltration is not enough to cause the involvement of multiple sites and vessels in a short time, so it is mostly manifested as type I.

Finally, our study found that changes in immunoglobulin in TAK patients in remission after treatment were not associated with improved disease activity. However, a decrease in IgG levels was still observed to be significantly associated with improvements in CRP and ESR. Among TAK patients in remission after treatment, the 1-year risk of disease recurrence was significantly higher in the immunoglobulin elevated group than in the immunoglobulin normal group. Notably, among the immunoglobulin subtypes, only the elevated IgG group had a significantly elevated 1-year recurrence risk; similar results were not observed in the elevated IgM and IgA groups. Previous studies found a mechanism by which IgG affected TAK patients. Jérôme Hadjadj et al. found that the mammalian target of rapamycin (mTOR) pathway was involved in TAK vascular remodeling. They found that mTORC1 and mTORC2 were explicitly activated in endothelial cells of TAK patients and confirmed that IgG purified from TAK patients could cause mTORC1 activation in endothelial cells, thus driving TAK endothelial remodeling [13]. Therefore, elevated IgG can be used as an indicator of disease activity in the TAK acute phase and as a reference indicator for disease remission.

There are limitations to our study. First, we are a single-center retrospective study, and the research methods and sample size may impact our results. Secondly, all TAK patients receive different strategies of systematic immunotherapy, which may lead to particular deviations. Finally, there were also patients lost to follow-up during our follow-up, which would also affect the results.

## Conclusion

Immunoglobulin is of clinical value in evaluating disease activity in TAK patients. Moreover, the dynamic changes in IgG were correlated with the changes of inflammatory markers in TAK patients. In TAK patients in remission, there was no difference in the risk of disease recurrence between the elevated immunoglobulin and the normal group.

## Data Availability

The datasets used and analyzed in the current study are available from the corresponding author on reasonable request.

## Abbreviations

TAK: Takayasu arteritis
IgG: Immunoglobulin G
IgM: Immunoglobulin M
IgA: Immunoglobulin A
IFN-γ: Interferon-γ
TNF-α: Tumor necrosis factor-α,
IL-2: Interleukin-2
IL-6: Interleukin-6
IL-12: Interleukin-12
IL-8: Interleukin-8
IL-17A: Interleukin-17A
IL-18: Interleukin-18
NIH: The National Institute of Health
ITAS: The Indian Takayasu Clinical Activity Score
BMI: Body mass Index
ESR: Erythrocyte sedimentation rate
CRP: C-reactive protein
BAFF: B cell survival factors activation factor
APRIL: A proliferation-inducing ligand
AECA: Anti-endothelial cell autoantibody
ACL: Anti-cardiolipin antibody
anti-β2GPI: β2 Glycoprotein I antibody
AAVA: Anti-annexin V antibody
mTOR: Mammalian target of rapamycin
